# Quantum Information Scrambling in Non-Markovian Open Quantum System

**DOI:** 10.3390/e24111532

**Published:** 2022-10-26

**Authors:** Li-Ping Han, Jian Zou, Hai Li, Bin Shao

**Affiliations:** 1 Key Laboratory of Advanced Optoelectronic Quantum Architecture and Measurement, Ministry of Education, School of Physics, Beijing Institute of Technology, Beijing 100081, China; 2 School of Science, Tianjin University of Technology, Tianjin 300384, China; 3 School of Information and Electronic Engineering, Shandong Technology and Business University, Yantai 264005, China

**Keywords:** information scrambling, non-Markovianity, non-Markovian quantum state diffusion (QSD) equation, tripartite mutual information (TMI), tripartite logarithmic negativity (TLN)

## Abstract

In this paper, we investigate the dynamics of a spin chain whose two end spins interact with two independent non-Markovian baths by using the non-Markovian quantum state diffusion (QSD) equation approach. Specifically, two issues about information scrambling in an open quantum system are addressed. The first issue is that tripartite mutual information (TMI) can quantify information scrambling properly via its negative value in a closed system, whether it is still suitable to indicate information scrambling in an open quantum system. We find that negative TMI is not a suitable quantifier of information scrambling in an open quantum system in some cases, while negative tripartite logarithmic negativity (TLN) is an appropriate one. The second one is that up to now almost all information scrambling in open quantum systems reported were focus on a Markovian environment, while the effect of a non-Markovian environment on information scrambling is still elusive. Our results show that the memory effect of an environment will be beneficial to information scrambling. Moreover, it is found that the environment is generally detrimental for information scrambling in the long-term, while in some cases it will be helpful for information scrambling in the short-term.

## 1. Introduction

Entanglement, as a key resource in quantum information processing, is believed to give significant insights into physical mechanisms in a variety of fields [1,2,3,4,5]. How quantum information, stored in local degrees of freedom in the initial state of a many-body system, propagates and distributes over the global degrees of freedom of the system, which is known as information scrambling, is an interesting topic from the fundamental point of view [6], and it stimulates a broad range of research interest in various fields, for example, quantum information [7,8], high energy physics [9,10], quantum-thermodynamics [11,12], condensed matter physics [13,14], etc. Information scrambling is generically identified as delocalization of quantum information [15,16,17,18] in a many-body system. A general accepted measure of information scrambling is the so-called out-of-time-order correlator (OTOC), which is associated with the growth of the square commutator between two initially commuting observables [15,19,20,21,22,23,24].

In addition to OTOC, tripartite mutual information (TMI) can also be a probe of information scrambling [7], which becomes negative if quantum information is delocalized, i.e., information is shared globally rather than in a bipartite manner. A particular advantage of TMI is that it does not rely on any selection of operators but only depends on the partitioning of the Hilbert space [25]. It has been proven that TMI is essentially equivalent to OTOC in capturing the feature of information scrambling by means of the channel–state duality [7], while it captures different aspects of quantum dynamics [26]. At first, TMI of the evolution operator was used to investigate information scrambling in References [27,28]. Later, instantaneous TMI of a quantum state was also used to study information scrambling in References [29,30,31]. The method used in this paper is instantaneous TMI of a quantum state. When TMI is non-negative at some time, the information at that moment is localized, while at some time when TMI is negative, the information is delocalized. If TMI is non-negative at the beginning and becomes negative as time evolves, the information gradually delocalizes, namely, information scrambling occurs. The definition of TMI is based on the von Neumann entropy, whose important caveat is that it captures both quantum and classical correlations. It is thus important to isolate the quantum contribution to the entropy. To this end, tripartite logarithmic negativity (TLN) [32] is analogously proposed to characterize the pure quantum information that is scrambled.

It is well-known that realistic quantum systems inevitably interact with their surrounding environments, resulting in decoherence and dissipation. The time evolution of such an open quantum system is usually characterized by a quantum master equation through Markovian approximation [33], corresponding to a memoryless environment, which leads to a monotonic information flow from the system of interest to the environment. When the environment’s memory cannot be ignored, a backflow of information from the environment to the system occurs, and the non-Markovian description of the system dynamics is required [34]. It has been found that non-Markovianity can lead to a significant variety of phenomena in the dynamics of open quantum systems [35,36,37,38,39,40,41] and can serve as a resource in information theory [42,43,44,45,46]. However, it is normally a hard task to solve non-Markovian dynamics of the system, and many theoretical approaches have been developed (see, e.g., References [47,48,49,50,51,52,53,54,55,56,57,58,59,60,61,62]). Among these approaches, the non-Markovian quantum state diffusion (QSD) equation method [48,49,50,51,52,53] has been proven to be effective.

It is noteworthy that information scrambling is rooted in the spread of entanglement, which is hard to preserve in the presence of an environment. The influence of environment noise on delocalization of information should not be neglected. Several works about open quantum system dynamics by using different quantifiers of information scrambling, such as corrected OTOC [63,64], a ratio of OTOC [65], mutual information [66], fidelity [67], etc., have been reported [63,64,65,66,67,68,69,70,71,72]. In Reference [64] it was found that taking an open bipartite OTOC as a probe, one can differentiate the contribution of information scrambling from decoherence and also distinguish integrable dynamics from chaotic ones. It was also found that dissipation and decoherence always suppress information scrambling for a Markovian environment [63,64]. It was shown in Reference [65] that one can distinguish information scrambling from decoherence in strongly interacting quantum systems by utilizing a teleportation-based decoding protocol. Touil and Deffner found that OTOC is not a good quantifier of information scrambling for open quantum systems, and they related the competing effects of information scrambling and decoherence to their respective contributions to the entropy change [66]. Up to now, most of the works about information scrambling by using TMI in the literature have focused on closed systems [3,25,26,29,30,73], while TMI for an open quantum system has not been fully considered. To our knowledge, there are so far three studies about information scrambling of open quantum systems by using TMI. In References [31] and [74] Y. Li et al. proposed a collision model to simulate the information dynamics in an all-optical system and found that non-Markovianity played dual roles in affecting the dynamics of information. In Reference [75], Sur and Subrahmanyam found that local quantum dynamical process can cause information scrambling even when the unitary evolution dynamics is non-scrambling in nature. Similar to OTOC, whether TMI is a suitable quantifier of information scrambling for an open quantum system is still an open question. Up to now, most of the information scrambling reported in open quantum systems have focused on a Markovian environment, the effect of non-Markovianity on information scrambling still being elusive and requiring further study.

To address these questions, in this paper we focus on information scrambling in the presence of an environment by using instantaneous TMI and TLN of a quantum state. The model we consider is a spin chain whose two end spins interact with two independent non-Markovian baths. We obtain the system’s dynamics by using the QSD equation approach. Interestingly, we find that in some cases, though TMI’s negative entanglement might be zero and thus negative TMI is not an appropriate probe of information scrambling in an open quantum system, negative TLN is. By comparing the dynamics of TLN with TMI, we can distinguish information scrambling from the total information delocalization in an open quantum system. Our results show that in general, environment is detrimental to information scrambling in the long-term, while in some cases environment will be helpful for the emergence of information scrambling in the short-term. More importantly, we find that non-Markovianity plays a beneficial role in keeping information scrambling.

This paper is organized as follows: In Section 2, we introduce the model and QSD equation approach which we use to solve non-Markovian dynamics of the system. In Section 3, we study the effect of baths on information scrambling for two types of system-bath interactions, i.e., dephasing and dissipation channels. In Section 4, we discuss the effects of non-Markovianity on information scrambling. In Section 5, we summarize our results. In the Appendix A, Appendix B and Appendix C, we show some supplemental results.

## 2. Model and Methods

The system we consider in this paper is a one-dimensional XXZ spin chain which consists of *N* qubits, and the Hamiltonian is

(1) $$H_{s}=\sum_{i=1}^{N-1}J_{i,i+1}\left(\sigma_{i}^{x}\sigma_{i+1}^{x}+\sigma_{i}^{y}\sigma_{i+1}^{y}+\Delta \sigma_{i}^{z}\sigma_{i+1}^{z}\right),$$

where $J_{i,i+1}$ is the coupling strength between the nearest neighbor sites *i* and i+1, and $\sigma^{j}\;\left(j=x,y,z\right)$ are the Pauli operators. Here, we take $J_{i,i+1}=-1$ throughout. When Δ=0, it is the non-interacting XX chain, which can be mapped to a free fermion model [76], and when Δ=1, it is the interacting XXZ spin chain, i.e., the isotropic Heisenberg chain, which is solvable by the Bethe ansatz [77]. The Hamiltonian Equation (1) is integrable, and the dynamics of such an integrable system can be understood by the propagation of quasi-particles, entangling different regions of the system as they propagate [78]. Information that is initially localized in some region is spread by these quasi-particles, which move at different velocities. Thus, information will disperse, leading in general to delocalized information among subsystems.

**Figure 1:**
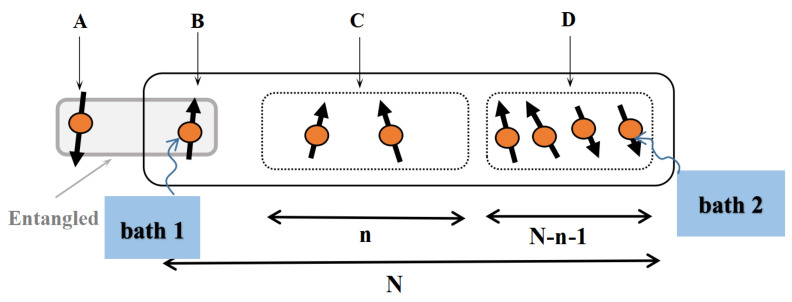
A schematic of the model considered in this paper. The spin chain is divided into three parts B, C and D, where its two end spins interact with two baths, i.e., bath 1 and bath 2, respectively. Qubit A is initially maximally entangled with qubit B, while C and D are not correlated with A and B initially. It is noticed that A is an ancillary qubit, which does not interact with the chain BCD.

We suppose that the two end spins of the chain interact with two baths $H_{1b}^{}$ and $H_{2b}^{}$, respectively (see Figure 1). The total Hamiltonian can be written as

(2) $$H_{\mathrm{tot}}=H_{s}+\sum_{j=1,2}H_{jb}+H_{\mathrm{int}},$$

with the free Hamiltonian for the left and right bosonic bath $H_{jb}^{}\;\left(j=1,2\right)$

(3) $$H_{jb}=\sum_{k}\omega_{jk}b_{jk}^{†}b_{jk},$$

and the interaction described by

(4) $$H_{\mathrm{int}}=\sum_{j=1,2}\sum_{k}\left(g_{jk}L_{j}b_{jk}^{†}+g_{jk}^{*}L_{j}^{†}b_{jk}\right).$$

Here, $L_{j}$ is the Lindblad operator, $b_{jk}^{†}\left(b_{jk}\right)$ is the bosonic creation (annihilation) operator of the kth mode of the jth bath with frequency $\omega_{jk}$, and $g_{jk}$ is the coupling strength between the system and the kth mode of the jth bath. A spin chain interacting with two baths independently at two ends, is widely used in the investigation of spin chain with open boundary condition, especially in the study of heat transport and quantum state transfer [79,80,81,82,83,84]. We assume that the baths are both at zero temperature, i.e., both baths are in the ground states $|0\rangle$ [85].

In order to investigate the effect of non-Markovianity on information scrambling, we use the QSD equation approach [49,53]. The basic idea is that the total wave function $|\Psi_{\mathrm{tot}}\left(t\right)\rangle$ is projected into the coherent state of the bath mode $|z\rangle$, and we have Ψz*(t)=z*z*Ψtot(t)Ψtot(t), which is known as stochastic quantum trajectory. It obeys a linear QSD equation [49,85]

(5) $$\begin{array}{c} \frac{\partial }{\partial t}|\Psi_{z^{*}}\left(t\right)\rangle =\left\{-iH_{s}+\sum_{j=1,2}\left[L_{j}z_{jt}^{*}-L_{j}^{†}{\bar{O}}_{j}\left(t,z^{*}\right)\right]\right\}|\Psi_{z^{*}}\left(t\right)\rangle , \end{array}$$

where $z_{jt}^{*}=-i\sum_{k}g_{jk}z_{jk}^{*}e^{i\omega_{jk}t}$ is a Gaussian stochastic process, *O* is an operator defined by $\frac{\delta }{\delta z_{js}^{*}}|\Psi_{z^{*}}\left(t\right)\rangle =O_{j}\left(t,s,z_{1}^{*},z_{2}^{*}\right)|\Psi_{z^{*}}\left(t\right)\rangle$, and ${\bar{O}}_{j}\left(t,z^{*}\right)=\int_{0}^{t}\alpha_{j}\left(t,s\right)O_{j}\left(t,s,z_{1}^{*},z_{2}^{*}\right)ds$. Assuming the bath is at zero temperature, the correlation function is $\alpha_{j}\left(t,s\right)=\sum_{k}{\left|g_{jk}\right|}^{2}e^{-i\omega_{jk}\left(t-s\right)}$ describing the effect of the bath, and $M\left[z_{jt}^{*}z_{js}^{}\right]=\alpha_{j}\left(t,s\right)$, where $M\left[\cdot \right]$ is the ensemble average.

According to the consistency condition, the *O* operator satisfies [85]

(6) $$\begin{array}{cc} \frac{\partial }{\partial t}O_{1}\left(t,s,z_{1}^{*},z_{2}^{*}\right)= & \left[-iH_{s}+\sum_{j=1,2}\left(L_{j}z_{jt}^{*}-L_{j}^{†}{\bar{O}}_{j}\left(t,z_{1}^{*},z_{2}^{*}\right)\right),O_{1}\left(t,s,z_{1}^{*},z_{2}^{*}\right)\right]\\ \; & -\sum_{j=1,2}L_{j}^{†}\frac{\delta }{\delta z_{2s}^{*}}{\bar{O}}_{j}\left(t,s,z_{1}^{*},z_{2}^{*}\right), \end{array}$$

(7) $$\begin{array}{cc} \frac{\partial }{\partial t}O_{2}\left(t,s,z_{1}^{*},z_{2}^{*}\right)= & \left[-iH_{s}+\sum_{j=1,2}\left(L_{j}z_{jt}^{*}-L_{j}^{†}{\bar{O}}_{j}\left(t,z_{1}^{*},z_{2}^{*}\right)\right),O_{2}\left(t,s,z_{1}^{*},z_{2}^{*}\right)\right]\\ \; & -\sum_{j=1,2}L_{j}^{†}\frac{\delta }{\delta z_{2s}^{*}}{\bar{O}}_{j}\left(t,s,z_{1}^{*},z_{2}^{*}\right). \end{array}$$

Instead of a direct numerical simulating the trajectories by the QSD equation above, we can analytically take the ensemble average to obtain a non-Markovian master equation. Based on Equations (6) and (7), the reduced density matrix of the system $\rho_{s}=M\left[P_{t}\right]$ can be obtained, where $P_{t}=|\Psi_{z^{*}}\left(t\right)\rangle \langle \Psi_{z}\left(t\right)|$. Using Novikov’s theorem [86,87,88], the general non-Markovian master equation can be derived as [89]

(8) $$\begin{array}{c} \frac{\partial }{\partial t}\rho_{s}=-i\left[H_{s},\rho_{s}\right]+\sum_{j=1,2}\left(\left[L_{j},M\left[P_{t}{\bar{O}}_{j}^{†}\right]\right]\right.-\left.\left[L_{j}^{†},\left.M\left[{\bar{O}}_{j}P_{t}\right]\right]\right.\right). \end{array}$$

It is noticed that the above equation is still not a closed equation for $\rho_{s}$. Generally, the operator ${\bar{O}}_{j}$ contains noises $z_{1}^{*}$, $z_{2}^{*}$. When ${\bar{O}}_{j}\left(t,z_{1}^{*},z_{2}^{*}\right)$ is approximated by a noise-independent operator, i.e., ${\bar{O}}_{j}\left(t,z_{1}^{*},z_{2}^{*}\right)={\bar{O}}_{j}\left(t\right)$, Equation (8) becomes a form of a time-local non-Markovian master equation [80]

(9) $$\begin{array}{cc} \frac{\partial }{\partial t} & \rho_{s}=-i\left[H_{s},\rho_{s}\right]+\sum_{j=1,2}\left(\left[L_{j},\rho_{s}{\bar{O}}_{j}^{†}\left(t\right)\right]\right.\left.-\left[L_{j}^{†},{\bar{O}}_{j}\left(t\right)\rho_{s}\right]\right). \end{array}$$

In the following, we will consider the correlation function $\alpha_{j}\left(t,s\right)=\frac{\Gamma_{j}\gamma_{j}}{2}e^{-\gamma_{j}\left|t-s\right|}$, which corresponds to the Ornstein–Uhlenbeck process [47,48,90,91]. Here, $\Gamma_{j}$ denotes the coupling strength between the system and the jth bath. $1/\gamma_{j}$ measures the correlation time between two separate time instances *t* and *s*, which indicates the memory time of the *j*th bath. When $\gamma_{j}$ is large enough, i.e., $1/\gamma_{j}$ is small enough, the dynamics can be approximately regarded as Markovian. When the parameter $\gamma_{j}$ is small, non-Markovian properties can be observed [85,92,93,94,95]. For the Ornstein–Uhlenbeck correlation ${\dot{\alpha }}_{j}(t,s)=-\gamma_{j}\alpha_{j}(t,s)$, the operator ${\bar{O}}_{1}\left(t\right)$ satisfies [80]

(10) $$\begin{array}{cc} \frac{\partial }{\partial t}{\bar{O}}_{1}\left(t\right) & =\frac{\Gamma_{1}\gamma_{1}}{2}L_{1}-\gamma_{1}{\bar{O}}_{1}\left(t\right)+\left[-iH_{s}-L_{1}^{†}{\bar{O}}_{1}\left(t\right)\right.\left.-L_{2}^{†}{\bar{O}}_{2}\left(t\right),{\bar{O}}_{1}\left(t\right)\right], \end{array}$$

(11) $$\begin{array}{cc} \frac{\partial }{\partial t}{\bar{O}}_{2}\left(t\right) & =\frac{\Gamma_{2}\gamma_{2}}{2}L_{2}-\gamma_{2}{\bar{O}}_{2}\left(t\right)+\left[-iH_{s}-L_{1}^{†}{\bar{O}}_{1}\left(t\right)\right.\left.-L_{2}^{†}{\bar{O}}_{2}\left(t\right),{\bar{O}}_{2}\left(t\right)\right], \end{array}$$

In this paper, we use the Runge–Kutta method to solve coupled Equations (9)–(11) numerically and then obtain the non-Markovian dynamics of the spin chain.

Next, we introduce the initial state used in this paper. Firstly, a product state between an ancillary qubit A and the system is prepared

(12) $$\begin{array}{c} \frac{1}{\sqrt{2}}\left({|0\rangle }_{A}+{|1\rangle }_{A}\right)⊗{|\Xi \rangle }_{BCD}. \end{array}$$

Here, ${|\Xi \rangle }_{BCD}$ is the initial state of system, which is divided into three parts B, C and D (see Figure 1). In this paper, the initial state of the system is chosen as a product state with the state of each qubit being $|0\rangle$ or $|1\rangle$ (e.g., the NÉEL state ${|\Xi \rangle }_{BCD}=|0101\ldots 01\rangle$, ${|\Xi \rangle }_{BCD}=|00\ldots 00\rangle$, etc.). Then, a CNOT gate is applied on qubit A and qubit B, and in this way the information about A is locally encoded in B through the entanglement between them.

In this paper, we will consider the following two types of Lindblad operators. The first type corresponds to $L_{j}=\sigma_{j}^{z}$(j=1,2), which describes the dephasing process. For this Lindblad operator, the z-component of the total spins in the system is a conserved quantity. The second type is $L_{j}=\sigma_{j}^{-}$(j=1,2), which describes the dissipative process, where $\sigma_{j}^{-}$ denotes the lowering operator.

## 3. Effects of Baths on Information Scrambling

In this section, we discuss the effects of baths on information scrambling for both dephasing and dissipation channels.

### 3.1. Initial NÉEL State

#### 3.1.1. Tripartite Mutual Information

We first consider TMI in the presence of baths for the initial NÉEL state. TMI among the ancillary qubit A and the subsystems B, C is defined as

(13) $$\begin{array}{c} I_{3}\left(A:B:C\right)=I_{2}\left(A:B\right)+I_{2}\left(A:C\right)-I_{2}\left(A:BC\right). \end{array}$$

$I_{2}\left(X:Y\right)=S_{X}+S_{Y}-S_{XY}$ is bipartite mutual information (BMI) between *X* and *Y*, which measures the total correlation (quantum and classical) between two subsystems of a composite system, and $S_{X}=-{\mathrm{Tr}}_{X}\left[{\hat{\rho }}_{X}\right.\left.\ln{\hat{\rho }}_{X}\right]$ is the von Neumann entropy of the corresponding reduced density matrix ${\hat{\rho }}_{X}$.

From an information–theoretic point of view, TMI quantifies how the total (quantum and classical) information is shared among the subsystems A, B and C. $I_{3}\left(A:B:C\right)$ is negative when $I_{2}\left(A:B\right)+I_{2}\left(A:C\right)<I_{2}\left(A:BC\right)$, which implies that the sum of the total information that is shared between A and B and A and C is smaller than that between A and BC together. In this case, the information about A is nonlocally stored in B and C such that measurements of B and C alone are not able to reconstruct A. Thus, a negative value of TMI is associated with delocalization of the total information, or the total information being scrambled. If TMI is non-negative at the beginning and becomes negative with time evolution, it means that information turns into delocalized; namely, the total information delocalization occurs.

**Figure 2:**
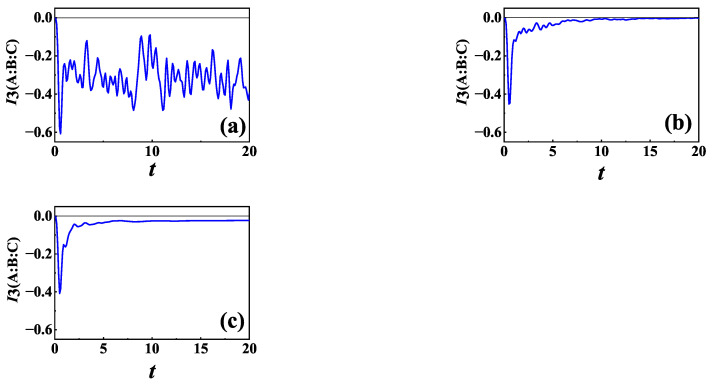
TMI of *XXZ* chain as a function of time for initial NÉEL state: (**a**) in the absence of bath (Γ=0); (**b**) $L=\sigma^{-}$; and (**c**) $L=\sigma^{z}$. For both (**b**,**c**), $\Gamma_{1}=\Gamma_{2}=\Gamma \;=\;0.5$, and $\gamma_{1}=\gamma_{2}=\gamma \;=\;5$. Here N=6, n=2.

We plot the time evolution of TMI for initial NÉEL state in Figure 2b,c for two different types of baths $L=\sigma^{-}$ and $L=\sigma^{z}$ with Γ=0.5, respectively, while Figure 2a is in the absence of baths (Γ=0) for comparison. It is shown in Figure 2a that TMI can be negative, implying that the total information (quantum and classical) is scrambled inside BCD in the absence of baths, which is consistent with the result of Reference [29]. Compared with Figure 2a, Figure 2b shows that the maximum absolute value of the negative value of TMI for $L=\sigma^{-}$ becomes smaller, and TMI gradually decays to zero in the presence of baths. It means that the total information is totally lost at last, and delocalization of the total information only lasts for a finite time. It can be seen from Figure 2c that the maximum absolute value of the negative value of TMI for $L=\sigma^{z}$ becomes smaller, and TMI decreases at first and finally arrives at a negative steady value. It is noted that the result is different from that of $L=\sigma^{-}$. More specifically, for $L=\sigma^{z}$ the information is not totally lost, and there is residual information at last. We calculate the entanglement between two arbitrary parts, i.e., A and B, A and C and A and BC by using bipartite logarithmic negativity (detailed definition is given in Section 3.1.2) and find that the entanglement has disappeared when TMI reaches its steady value, which means that in this case there is no more quantum correlation, let alone quantum information scrambling. As is known, information scrambling is related to quantum correlation, and from the above results, we can learn that negative TMI does not always mean information scrambling for an open quantum system because the residual information at last is purely classical in this case. The different results for initial NÉEL state between $L=\sigma^{-}$ and $L=\sigma^{z}$ can be understood as follows. For $L=\sigma^{z}$, the off-diagonal elements of the density matrix of the system gradually decay with time evolution and disappear at last. In this case, though entanglement disappears, classical correlations still can exist at last. Different from $L=\sigma^{z}$, for $L=\sigma^{-}$ the system gradually decays to ground state with time evolution. In this case, both entanglement and classical correlations disappear at last, and thus TMI finally decays to zero.

It is noticed that for a closed system with limited dimension, the dynamics of a system usually show oscillatory behaviors. In this paper, we focus on the effects of baths on information scrambling. As shown in Figure 2a, without the baths TMI exhibits oscillatory behaviors as expected, while TMI will gradually decay in the presence of baths as shown in Figure 2b,c. From Figure 2a–c, it can also be seen that the presence of baths does not change the position of the peaks and valleys of TMI.

#### 3.1.2. Tripartite Logarithmic Negativity

An important caveat of the von Neumann entropy is that it captures both quantum and classical correlations. Then, it is necessary to isolate the quantum contribution. To this end, we consider the bipartite logarithmic negativity (BLN), which is a proper measure of entanglement in the mixed state, and its definition is [96]

(14) $$\begin{array}{c} \varepsilon_{2}=\log\left({\left|\rho_{XY}^{T_{Y}}\right|}_{1}\right), \end{array}$$

where $\rho_{XY}^{T_{Y}}$ is the partial transpose of a density matrix, and ${\left|\rho_{XY}^{T_{Y}}\right|}_{1}=\mathrm{Tr}\sqrt{{\left(\rho_{XY}^{T_{Y}}\right)}^{†}\rho_{XY}^{T_{Y}}}$ is the trace norm of $\rho_{XY}^{T_{Y}}$. By replacing BMI on the right side of Equation (13) with BLN, analogous to the quantity TMI, TLN is defined as [32]

(15) $$\begin{array}{c} \varepsilon_{3}\left(A:B:C\right)=\varepsilon_{2}\left(A:B\right)+\varepsilon_{2}\left(A:C\right)-\varepsilon_{2}\left(A:BC\right). \end{array}$$

A negative value of TLN implies delocalization of quantum information among A, B and C, while a non-negative value of TLN indicates that in this case quantum information mostly is stored in bipartite partitions and is not delocalized.

**Figure 3:**
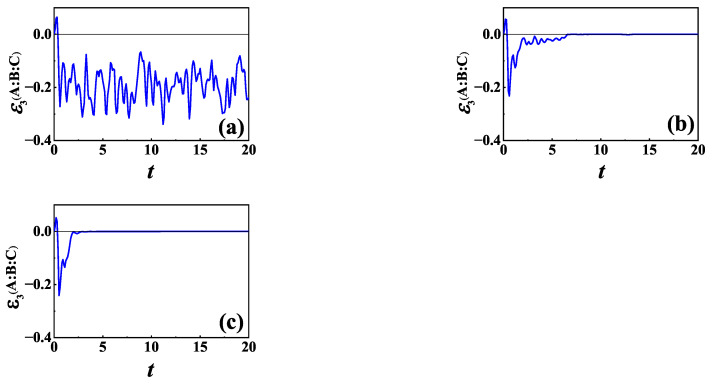
TLN of XXZ chain as a function of time for initial NÉEL state: (**a**) in the absence of bath (Γ=0); (**b**) $L=\sigma^{-}$; and (**c**) $L=\sigma^{z}$. All the parameters are the same as those in Figure 2.

In Figure 3, we plot the time evolution of TLN for the same initial NÉEL state as in Figure 2. Figure 3a shows the time evolution of TLN in the absence of baths (Γ=0) for comparison, while Figure 3b,c are for $L=\sigma^{-}$ and $L=\sigma^{z}$ with Γ=0.5, respectively. The behavior of TLN shown in Figure 3 is similar to that in Figure 2. TLN in Figure 3a can be negative, which indicates that the quantum information is also scrambled in the absence of baths. Comparing Figure 3b,c with Figure 3a, we can see that the maximum absolute value of the negative value of TLN becomes smaller,and the duration of delocalization of quantum information is limited.

Unlike TMI for $L=\sigma^{z}$ saturating to a negative steady value after a long time evolution, TLN (see Figure 3c) decreases to zero at last, which means that finally quantum information is totally lost. Comparing Figure 2 with Figure 3, we can see that TMI lasts for a longer time than TLN in the presence of baths. Especially for $L=\sigma^{z}$, TMI saturates to a negative value after TLN decays to zero. It implies that when entanglement is zero, TMI can still be negative. Hence, negative TMI is not a good diagnosis of information scrambling for open quantum systems. By comparing the dynamics of TLN and TMI, we can distinguish information scrambling from the total information delocalization in an open quantum system. Therefore, in the following, we will focus on TLN.

The decay of TLN to zero at last in the presence of baths shown in Figure 3b,c implies that information scrambling is suppressed by these two different types of baths. Information scrambling can only occur in the short-term, and then disappears in a long-term. This phenomenon can be understood as the interaction between the system and baths creating entanglement between them, which in turn destroys the entanglement within the system, and hence diminishes delocalization of quantum information. It is noticed that though the environment has a negative impact on information scrambling, there are regimes in which quantum information is still scrambled in the early period.

Comparing these two types of system–bath interactions, it can be seen that the time interval that TLN stays negative in the case of $L=\sigma^{-}$ is larger than that in the case of $L=\sigma^{z}$ for the same values of Γ and γ. For $L=\sigma^{z}$, the total number of excitations for both ancillary qubit A and the system is conserved; thus, the effective Hilbert subspace for quantum information is the same as that without baths. It is noticed that for $L=\sigma^{-}$ and initial NÉEL state, at the beginning due to partially decaying, the space belonging to each excitation might be occupied, which means that the effective Hilbert subspace is enlarged at the early time. As time evolves further, the number of excitations gradually decreases, and at last, the system will evolve into the ground state completely, i.e., the size of effective Hilbert subspace after a transient period of time is gradually decreased. Although the total number of excitations is conserved in the case of $L=\sigma^{z}$, decoherence occurs, which means that the coherence and quantum correlation gradually disappear as time evolves, in which case $L=\sigma^{z}$ or $L=\sigma^{-}$ information scrambling lasts for a longer time, depending on which one decays faster, the coherence or the excitation. For XXZ chain, the coherence decays faster for $L=\sigma^{z}$ than the excitation decays for $L=\sigma^{-}$.

### 3.2. Initial State $|00\ldots 00\rangle$

**Figure 4:**
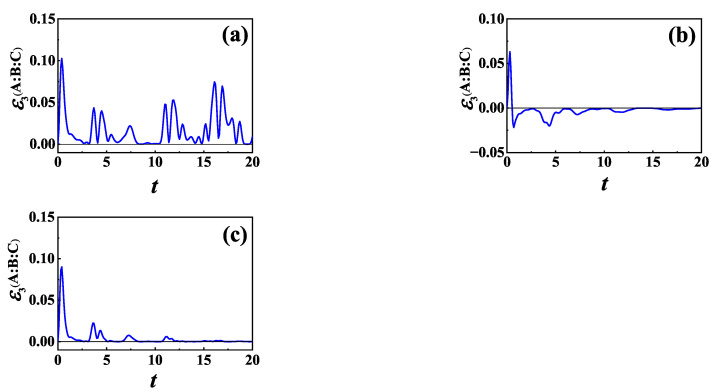
TLN of XXZ chain as a function of time for initial state $|00\ldots 00\rangle$: (**a**) in the absence of bath (Γ=0); (**b**) $L=\sigma^{z}$; and (**c**) $L=\sigma^{-}$. Here, N=7, n=1 and the other parameters are the same as those in Figure 2.

Next, we consider the initial state $|00\ldots 00\rangle$. Figure 4a shows the time evolution of TLN in the absence of baths (Γ=0), while Figure 4b,c are for $L=\sigma^{z}$ and $L=\sigma^{-}$, respectively. As shown in Figure 4a, TLN is non-negative without baths for this initial state, implying that quantum information is not scrambled. The reason quantum information is not scrambled for this initial state in the unitary case is that there is only one excitation for this initial state; thus, there are few quasi-particles [97], which confines the dynamics and hence constrains the amount of entanglement that can emerge. Accordingly, quantum information is stored mostly in bipartite partitions and cannot spread properly over many degrees of freedom.

In contrast, in Figure 4b, TLN in the presence of dephasing baths can become slightly negative, which means that information scrambling takes place. On the other hand, after a long time evolution, TLN decays to zero as shown in Figure 4b. It indicates that quantum information is totally lost at last, and information scrambling can only exist for a short time. Completely different from the results for channel $L=\sigma^{z}$, TLN still stays non-negative for channel $L=\sigma^{-}$ shown in Figure 4c, which means that information scrambling does not occur. We will explain the reason in the following subsection.

### 3.3. A Class of Initial Product States

**Figure 5:**
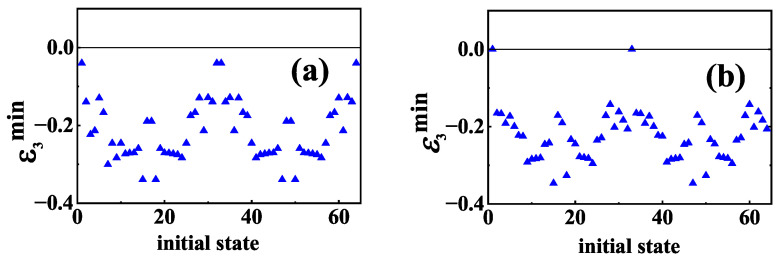
Initial-state dependence of minimum values of TLN in the case of: (**a**) $L=\sigma^{z}$ and (**b**) $L=\sigma^{-}$ respectively. The horizontal axis shows the labels of initial states in a decimal. All the parameters are the same as those in Figure 2.

We have studied the effects of baths on information scrambling for two specific initial states. Now, we consider a class of initial product states. We consider all of the $2^{N}$ permutations on each qubit state being $|0\rangle$ or $|1\rangle$ as initial states, ${|\Xi \rangle }_{BCD}$, and label these $2^{N}$ product states by bit sequences from $|11\ldots 11\rangle$ to $|00\ldots 00\rangle$. We investigate the initial-state dependence of information scrambling by using minimum values of TLN. The reason why we choose minimum values of TLN is that they can be used to describe how strong information scrambling is. Figure 5 displays the initial-state dependence of minimum values of TLN, written as $\varepsilon_{3}^{\min}$, for (a) dephasing channel and (b) dissipation channel, respectively. The horizontal axis shows the labels of ${|\Xi \rangle }_{BCD}$ in a decimal. From Figure 5, we can see that for $L=\sigma^{z}$ information scrambling occurs ($\varepsilon_{3}^{\min}<0$) for all these initial product states, including the four initial states for which information scrambling cannot occur in the absence of baths, i.e., $|00\ldots 00\rangle$, $|10\ldots 00\rangle$, $|01\ldots 11\rangle$ and $|11\ldots 11\rangle$ [29]. Similar to the case for $|00\ldots 00\rangle$, because the size of effective Hilbert subspace is too small, quantum information cannot be scrambled for initial states $|10\ldots 00\rangle$, $|01\ldots 11\rangle$ and $|11\ldots 11\rangle$ in the absence of baths. Information scrambling can occur for these four initial states in the presence of dephasing baths, which can be understood as the system–bath interaction destroys the quasi-particle and thus changes the localized dynamics to a delocalized one.

It can also be seen from Figure 5 that for $L=\sigma^{-}$, except for $|00\ldots 00\rangle$ and $|10\ldots 00\rangle$, information scrambling can occur for all the other initial states. The different results for $|00\ldots 00\rangle$ and $|10\ldots 00\rangle$ between $L=\sigma^{z}$ and $L=\sigma^{-}$ can be understood as the different sizes of their corresponding effective Hilbert subspaces. It is noticed that due to the use of CNOT gate, for these two initial states, the total number of excitations for both ancillary qubit A and the system is one. For $L=\sigma^{z}$, as mentioned above, the total number of excitations is conserved; thus, the effective Hilbert subspace for quantum information is not decreased. However, for $L=\sigma^{-}$, the total number of excitations for both ancillary qubit A and the system is gradually decreased from 1 to 0; thus, the effective Hilbert subspace for quantum information is always decreased. Hence, the amount of entanglement that can emerge is severely limited, and information scrambling cannot occur for $L=\sigma^{-}$. We also notice that similar to the case for $L=\sigma^{-}$ with initial NÉEL state, for $|01\ldots 11\rangle$ and $|11\ldots 11\rangle$, the effective Hilbert subspace is enlarged for a while before it starts to decrease; thus, information scrambling occurs for these two states. In a word, information scrambling can occur for all these $2^{N}$ initial product states in the case of $L=\sigma^{z}$, while in the case of $L=\sigma^{-}$, information scrambling can still occur for all these initial product states except $|00\ldots 00\rangle$ and $|10\ldots 00\rangle$.

## 4. Effects of Non-Markovianity on Information Scrambling

In the following, we will investigate the effects of non-Markovianity on information scrambling. As discussed above for our model, information scrambling can occur for most of these initial product states in the absence of baths. We first consider the initial states with which information scrambling can occur in the absence of baths. Our investigation shows that the effects of non-Markovianity on information scrambling for these initial states are qualitatively the same. In the following, we take initial NÉEL state as an example.

**Figure 6:**
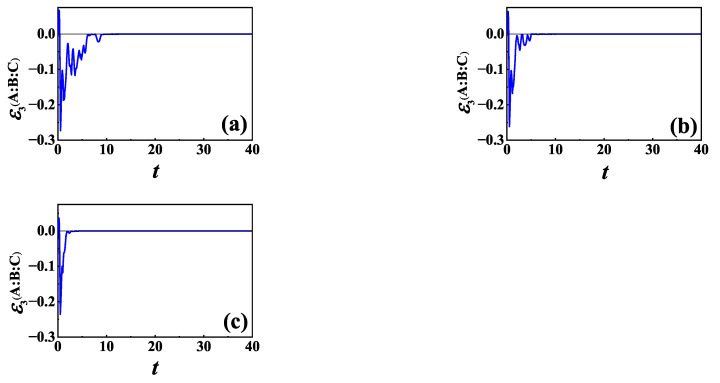
TLN of XXZ chain versus time *t* in the case of $L=\sigma^{z}$ for initial NÉEL state and different γ: (**a**) γ=1; (**b**) γ=2; and (**c**) γ→∞. The other parameters are Γ=0.5, N=6 and n=2.

For $L=\sigma^{z}$, we plot the time evolution of TLN for initial NÉEL state and different γ in Figure 6, for (a) γ=1, (b) γ=2 and (c) γ→∞, respectively. Clearly, the presence of the baths will suppress information scrambling. However, it can be seen from Figure 6 that with the decrease of γ, i.e., the increase of non-Markovianity, the maximum absolute value of the negative value of TLN increases, and it takes more time for TLN to decay to zero.

It is known that γ indicates a memory effect of the environment, and the smaller the γ, the longer the environmental memory time. When γ is small enough, non-Markovian properties can be observed. It has been shown that non-Markovianity due to the information backflow can be traced back to the establishment of correlations between the system and the environment as well as the change in the state of the environment [98,99,100]. In the Markovian case, information of the system flows completely into the environment. While in the non-Markovian case, information flowing from the system is partially preserved during the transient period in the correlation between the system and the environment as well as in the environment and will subsequently flow back to the system. From Figure 6, we can find that with the decrease of γ, the oscillation lasts longer and decays more slowly for TLN. Thus, information scrambling lasts for a longer time in non-a case than in a Markovian case.

**Figure 7:**
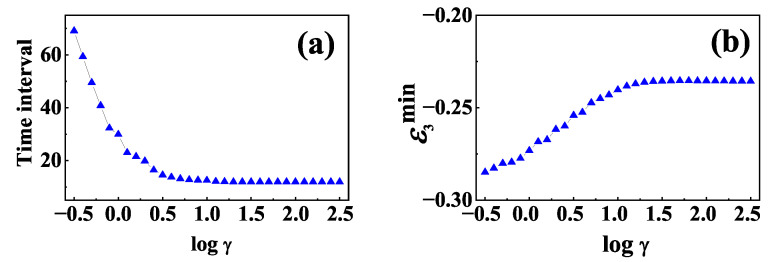
(**a**) The time interval TLN staying negative as a function of logγ; (**b**) The minimum value of TLN as a function of logγ. The initial state and the other parameters are the same as those in Figure 6.

To make the results for the effects of non-Markovianity on information scrambling more quantitative, we plot the time interval TLN staying negative and the minimum value of TLN as functions of logγ in Figure 7a,b, respectively. It is shown in Figure 7a that the time interval TLN staying negative decreases to a steady value with the increase of γ, and it is longer for non-Markovian baths than that for Markovian ones. On the other hand, from Figure 7b we can find that the minimum value of TLN increases as γ increases, and it is smaller for non-Markovian baths than that for Markovian baths. These results suggest that baths with memory will be beneficial to the emergence of information scrambling.

**Figure 8:**
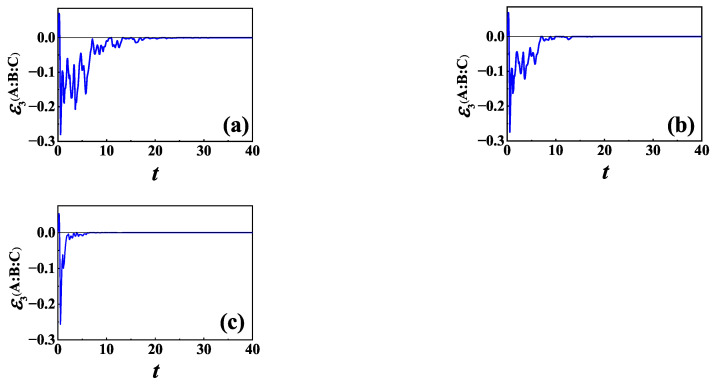
TLN of XXZ chain versus time *t* in the case of $L=\sigma^{-}$ for initial NÉEL state and different γ: (**a**) γ=1; (**b**) γ=2; and (**c**) γ→∞. The other parameters are the same as those in Figure 6.

Now, we consider $L=\sigma^{-}$. In Figure 8, we plot the time evolution of TLN for different γ for the same initial NÉEL state as in Figure 6. From numerical calculation, we find that the memory effect of the baths is helpful for information scrambling in the case of $L=\sigma^{-}$ also, which is qualitatively the same as that for $L=\sigma^{z}$ shown in Figure 6.

**Figure 9:**
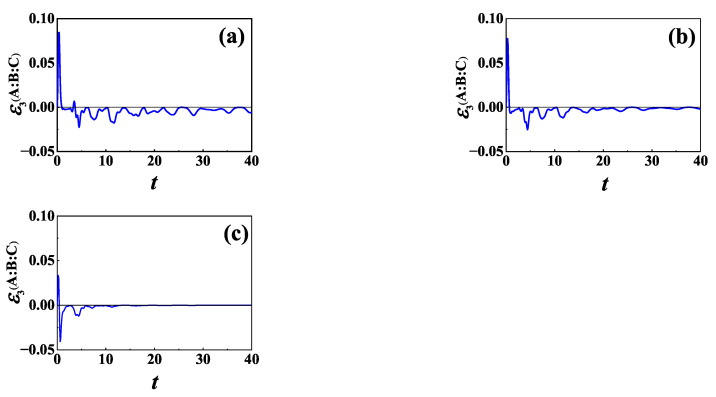
TLN of XXZ chain versus time *t* in the case of $L=\sigma^{z}$ for initial state $|00\ldots 00\rangle$ and different γ: (**a**) γ=1; (**b**) γ=2; (**c**) γ→∞. The other parameters are Γ=0.5, N=7 and n=1.

We then consider the initial states with which information scrambling cannot occur in the absence of baths. For these states, it is found that the effects of non-Markovianity on information scrambling are similar, so we take $|00\ldots 00\rangle$ as an example. As mentioned above, for this initial state in the case of $L=\sigma^{z}$, system–bath interaction can change the localized dynamics to a delocalized one in the early period, while information scrambling cannot occur for this initial state in the case of $L=\sigma^{-}$ whether in Markovian or non-Markovian regimes. Figure 9 shows the time evolution of TLN for $L=\sigma^{z}$ and different γ. It can be seen from Figure 9 that the time interval TLN stays negative decreases with the increase of γ, and it is longer for non-Markovian baths than that for Markovian ones, which are similar to those for initial NÉEL state. This indicates that baths with memory can also enhance information scrambling for these initial states.

## 5. Conclusions

In this paper, we have studied information scrambling by using tripartite mutual information and tripartite logarithmic negativity. We have considered a spin chain with two ends interacting with two separate baths and used the non-Markovian quantum state diffusion equation approach to obtain the time evolutions of TMI and TLN. We have considered two types of system–bath interactions, i.e., dephasing and dissipation channels as well as various initial product states.

It has been found that TMI can still be negative when there is no entanglement at all, which means that negative TMI might not be a suitable quantifier of information scrambling for an open quantum system anymore, but negative TLN is an appropriate one. By comparing the dynamics of TLN with TMI, we can distinguish information scrambling from the total information delocalization in an open quantum system.

Our results have shown that generally the existence of baths suppresses information scrambling in the long-term. However, in some cases environment can play a beneficial role. For example, for the initial state $|00\ldots 00\rangle$, information is not scrambled in the absence of baths, while information scrambling can occur in the early period with dephasing baths. These phenomena can be understood as that the system–bath interaction destroys the quasi-particle and thus changes the localized dynamics to a delocalized one. More importantly, it has been found that non-Markovianity can be helpful for keeping information scrambling. Concretely, information scrambling lasts longer in non-Markovian regime than that in Markovian regime, and with the increase of non-Markovianity, information scrambling lasts longer and longer.

In addition, we also considered the non-interacting spin chain (Δ=0, XX chain). From numerical calculations, we found that the results for TMI and TLN are only slightly different from those for the XXZ chain (for detail see Appendix A). In addition, we considered the influences of the size of subsystem C and the system–bath interaction strength Γ on information scrambling in the presence of baths. We found that with the increase in size of C, information scrambling lasts a longer time for the XXZ chain (see Appendix B), and we found that the maximum absolute value of the negative value for TLN as well as the time duration before it decays to zero decrease with the increase of Γ, which implies that a stronger system–bath interaction corresponds to less information scrambling (see Appendix C).

## Figures and Tables

**Figure A1:**
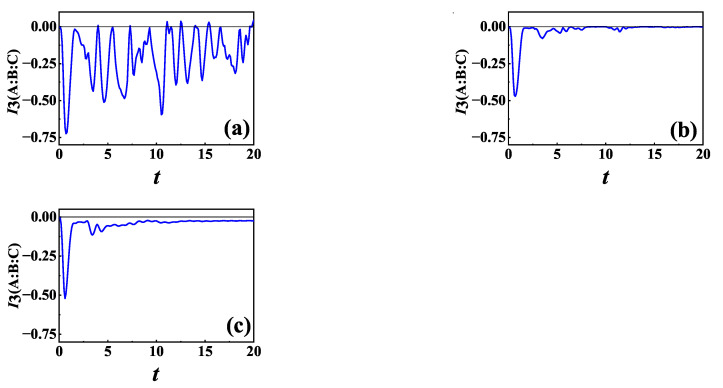
TMI of XX chain as a function of time for initial NÉEL state: (**a**) in the absence of bath (Γ=0); (**b**) $L=\sigma^{-}$; (**c**) $L=\sigma^{z}$. For both (**b**,**c**), $\Gamma_{1}=\Gamma_{2}=\Gamma \;=\;0.5$ and $\gamma_{1}=\gamma_{2}=\gamma \;=\;5$. Here, N=6, n=2.

**Figure A2:**
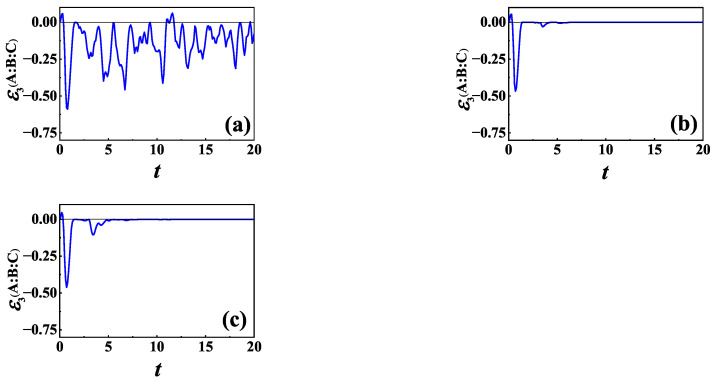
TLN of XX chain as a function of time for initial NÉEL state: (**a**) in the absence of bath (Γ=0); (**b**) $L=\sigma^{-}$; (**c**) $L=\sigma^{z}$. All the parameters are the same as those in Figure A1.

**Figure A3:**
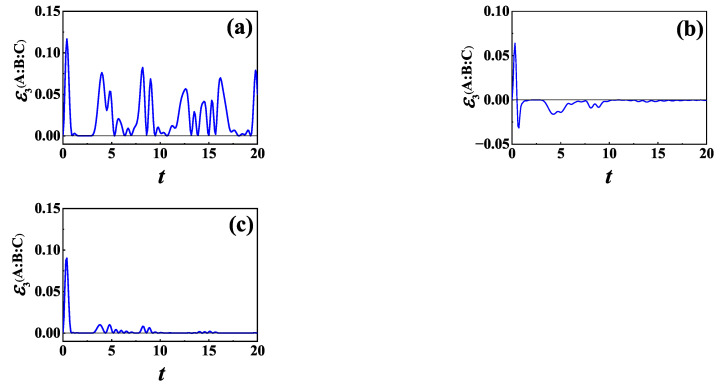
TLN of XX chain as a function of time for initial state $|00\ldots 00\rangle$: (**a**) in the absence of bath (Γ=0); (**b**) $L=\sigma^{z}$; (**c**) $L=\sigma^{-}$. Here, N=7, n=1, and the other parameters are the same as those in Figure A1.

**Figure A4:**
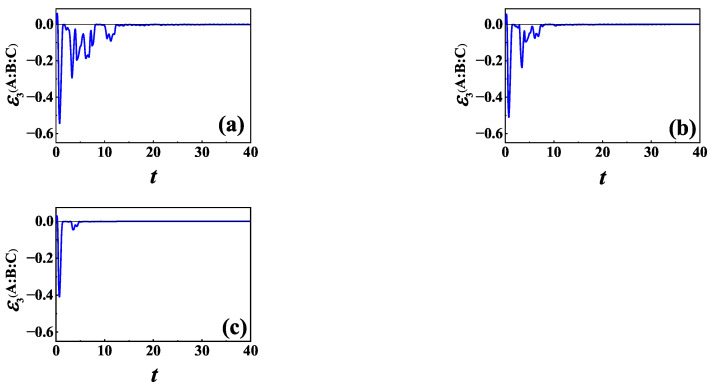
TLN of XX chain versus time *t* in the case of $L=\sigma^{z}$ for initial NÉEL state and different γ: (**a**) γ=1; (**b**) γ=2; (**c**) γ→∞. The other parameters are Γ=0.5, N=6 and n=2.

**Figure A5:**
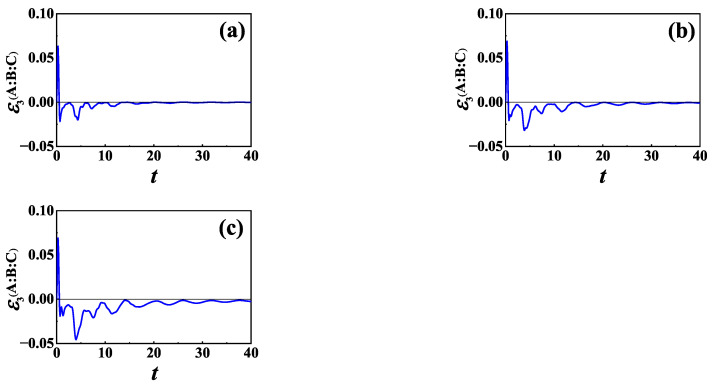
TLN of XXZ chain versus time *t* in the case of $L=\sigma^{z}$ for initial state $|00\ldots 00\rangle$ and different *n*: (**a**) n=1; (**b**) n=2; (**c**) n=3. The other parameters are N=7, Γ=0.5 and γ=5.

**Figure A6:**
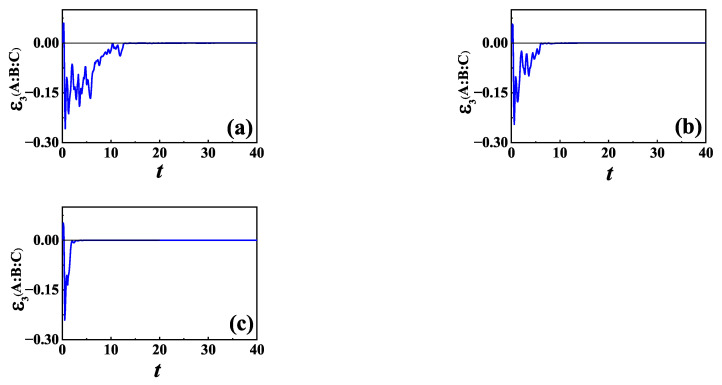
TLN of XXZ chain versus time *t* in the case of $L=\sigma^{z}$ for initial NÉEL state and different Γ: (**a**) Γ=0.1; (**b**) Γ=0.2; (**c**) Γ=0.5. The other parameters are N=6, γ=5 and n=2.

## Data Availability

Data sharing not applicable.

## Appendix A. The Numerical Results for *XX* Chain

In this Appendix, we show the numerical results for the XX non-interacting spin chain (Δ=0). For the XX chain, the results for TMI and TLN are only slightly different from those for the XXZ chain. From Figure A1 and Figure A2, we can see that TMI and TLN for the initial NÉEL state are both suppressed in the presence of baths, which are qualitatively the same as those for the XXZ chain. However, for two different types of system–bath interactions, the result is different from that for the XXZ chain. From Figure A2b,c, we can see that the time interval that TLN stays negative in the case of $L=\sigma^{z}$ is longer than that in the case of $L=\sigma^{-}$, which is different from the result for the XXZ chain shown in Figure 3b,c. It indicates that for the XX chain, the excitation decays faster for $L=\sigma^{-}$ than the coherence decays for $L=\sigma^{z}$. The detrimental effect of the dephasing channel is weaker than that of the dissipation channel for the XX chain for keeping information scrambling.

In Figure A3, we plot TLN as a function of time *t* for initial state $|00\ldots 00\rangle$. The results show that information scrambling can also occur for $L=\sigma^{z}$, while for this initial state it cannot occur for $L=\sigma^{-}$, which are the same as those for the XXZ chain shown in Figure 4.

Taking the initial NÉEL state as an example, the benefits of the memory effect of $L=\sigma^{z}$ on the emergence of information scrambling can also be seen in Figure A4, which is consistent with the result shown in Figure 6. In the case of $L=\sigma^{-}$, the effect of non-Markovianity is also helpful for keeping information scrambling, which is also consistent with the result for the XXZ chain.

For the initial state $|00\ldots 00\rangle$, from numerical calculation it is found that the effect of non-Markovianity is also beneficial for keeping information scrambling in the case of $L=\sigma^{z}$. While for $L=\sigma^{-}$, information scrambling cannot occur whether in Markovian or non-Markovian regimes. These results are qualitatively the same as those for the XXZ chain.

## Appendix B. The Influence of the Size of C on TLN for the *XXZ* Chain

Then, we consider the influence of the size of C on TLN for the XXZ chain. In Figure A5, we plot TLN as a function of time *t* for different *n* and initial state $|00\ldots 00\rangle$ in the case of $L=\sigma^{z}$. It can be seen from Figure A5 that with the increase of the spin number of subsystem C, the maximum absolute value of the negative value for TLN becomes larger, and it takes more time for TLN to decay to zero. In a word, with the increase in size of C, information scrambling can last for a longer time, and more information is scrambled. For the initial NÉEL state, the results are qualitatively the same as those for initial state $|00\ldots 00\rangle$.

In the case of $L=\sigma^{-}$, as mentioned above information scrambling cannot occur for the initial state $|00\ldots 00\rangle$ or $|10\ldots 00\rangle$ and except for these two states, it also takes more time for TLN to decay to zero for the XXZ chain with the increase of the spin number *n* of subsystem C.

## Appendix C. The Effects of Γ on TLN for *XXZ* Chain

In this Appendix, we investigate the effects of Γ on TLN. In Figure A6, we plot TLN versus time *t* for initial NÉEL state and different Γ in the case of $L=\sigma^{z}$. From Figure A6, it can be seen that the maximum absolute value of the negative value for TLN as well as the time duration before it reaches zero decreases with increasing Γ, which implies that a stronger system–bath interaction corresponds to less quantum information scrambling. The effect of Γ on TLN for $L=\sigma^{-}$ is similar to that for $L=\sigma^{z}$.
